# Circ_0004104 knockdown alleviates oxidized low-density lipoprotein-induced dysfunction in vascular endothelial cells through targeting miR-328-3p/TRIM14 axis in atherosclerosis

**DOI:** 10.1186/s12872-021-02012-7

**Published:** 2021-04-23

**Authors:** Chi Zhang, Liyue Wang, Ying Shen

**Affiliations:** Department of Cardiology, The Puren Hospital, No. 218, Changqing First Road, Jianghan District, Wuhan, 430081 Hubei China

**Keywords:** Atherosclerosis, Oxidized low-density lipoprotein, Circ_0004104, MiR-328-3p, TRIM14

## Abstract

**Background:**

Circular RNAs have shown important regulatory roles in cardiovascular diseases, containing atherosclerosis (AS). We intended to explore the role of circ_0004104 in AS using oxidized low-density lipoprotein (ox-LDL)-induced vascular endothelial cells and its associated mechanism.

**Methods:**

Real-time quantitative polymerase chain reaction and Western blot assay were conducted to analyze RNA levels and protein levels, respectively. Cell viability, apoptosis, angiogenic ability and inflammatory response were assessed by 3-(4,5-Dimethylthiazol-2-yl)-2,5-Diphenyltetrazolium Bromide (MTT) assay, flow cytometry, capillary-like network formation assay and enzyme-linked immunosorbent assay, respectively. Cell oxidative stress was assessed using commercial kits. Dual-luciferase reporter assay, RNA immunoprecipitation assay and RNA-pull down assay were performed to verify the intermolecular interaction.

**Results:**

ox-LDL exposure up-regulated the level of circ_0004104 in HUVECs. ox-LDL exposure suppressed cell viability and angiogenic ability whereas promoted the apoptosis, inflammation and oxidative stress of HUVECs partly through up-regulating circ_0004104. MicroRNA-328-3p (miR-328-3p) was confirmed as a target of circ_0004104. MiR-328-3p interference largely reversed circ_0004104 silencing-mediated effects in HUVECs upon ox-LDL exposure. MiR-328-3p interacted with the 3′ untranslated region of tripartite motif 14, and circ_0004104 positively regulated TRIM14 expression by sponging miR-328-3p. TRIM14 overexpression largely overturned miR-328-3p accumulation-induced influences in HUVECs upon ox-LDL exposure.

**Conclusion:**

Circ_0004104 knockdown attenuated ox-LDL-induced dysfunction in HUVECs via miR-328-3p-mediated regulation of TRIM14.

**Supplementary Information:**

The online version contains supplementary material available at 10.1186/s12872-021-02012-7.

## Background

Many types of cells are involved in the pathogenetic process of atherosclerosis (AS), containing vascular endothelial cells and smooth muscle cells [1]. Endothelial injury theory is one of the mainstream theories of atherosclerosis pathogenesis, and it considers artery atheromatous plaque as the product of endothelial injury [2]. Oxidized low-density lipoprotein (ox-LDL) is a crucial risk factor that is responsible for AS initiation [3]. Therefore, we established AS cell model using ox-LDL-treated HUVECs to find the pivotal molecules that were involved in AS pathogenesis in vitro.

Circular RNAs (circRNAs) are endogenous RNAs without 5′ or 3′ polarity [4, 5]. CircRNAs have been demonstrated to modulate the pathological process of human malignancies [6, 7]. Also, accumulating studies have identified the important roles of circRNAs in AS. For instance, Li et al. found that circ_0003575 knockdown accelerated the proliferation ability and tube formation capacity of HUVECs [8]. Liu et al. demonstrated that circ_0003204 suppressed proliferation and angiogenesis of ox-LDL-treated HUVECs [9]. Circ_0004104 was reported to be notably up-regulated in patients diagnosed with coronary artery disease [10]. However, the role and mechanism of circ_0004104 in AS progression remain largely unknown.

MicroRNAs (miRNAs) reversely modulate gene expression by interacting with the 3′ untranslated region (3′UTR) of messenger RNAs (mRNAs), causing translational repression or degradation of mRNAs [11, 12]. Dysregulation of miRNAs was associated with AS progression [13]. We concentrated on the biological significance of miR-328-3p in AS progression, which was predicted to be a candidate downstream miRNA of circ_0004104 by bioinformatic database. Guo et al. claimed that miR-328-3p attenuated ox-LDL-mediated dysfunction in HUVECs [14]. Nevertheless, the working mechanism of miR-328-3p in AS still needs to be further clarified.

Tripartite motif 14 (TRIM14) is one of the members of TRIM family [15]. TRIM14 exerted an oncogenic role in many human malignancies [16–19]. In addition, Huang et al. demonstrated that TRIM14 could accelerate the activation of endothelium through activating NF-κB signaling [20]. TRIM14 was predicted by bioinformatic database to be a downstream gene of miR-328-3p, and the working mechanism of TRIM14 in AS progression was investigated.

We initially explored the role of circ_0004104 in AS cell model. Subsequently, the working mechanism of circ_0004104 was explored through bioinformatic analysis and rescue experiments.

## Methods

### Cell line

Human umbilical vein endothelial cells (HUVECs) acquired from Chinese Academy of Medical Sciences, Shanghai institute Cell Bank (Shanghai, China) were cultivated in Dulbecco’s modified Eagle’s medium (DMEM; Gibco, Carlsbad, CA, USA) plus 10% fetal bovine serum (FBS, Hyclone, Carlsbad, CA, USA) and 1% antibiotics (Gibco) under 37℃ humidified atmosphere with 5% CO_2_.

### AS cell model

HUVECs were exposed to 100 μg/mL ox-LDL (Solarbio, Beijing, China) for 24 h to establish AS cell model as previously reported [8, 14].

### Real-time quantitative polymerase chain reaction (RT-qPCR)

RNA samples were isolated using Trizol reagent (Invitrogen, Carlsbad, CA, USA). Complementary DNA (cDNA) was synthesized using the miScript Reverse Transcription kit (for miRNA; Qiagen, Valencia, CA, USA) and RevertAid First Strand cDNA Synthesis Kit (for circRNA and mRNA; Invitrogen). cDNA was amplified via the SYBR™ Green PCR Master Mix (Invitrogen). The primers purchased from Sangon Biotech (Shanghai, China) were shown in Table 1. Relative abundance of circ_0004104, SPARC and TRIM14 was analyzed using the 2^−ΔΔCt^ method with glyceraldehyde-3-phosphate dehydrogenase (GAPDH) as reference, while the fold change of miR-328-3p was calculated using the 2^−ΔΔCt^ method with U6 as reference.

**Table 1:** Specific primers in RT-qPCR assay

Gene	Direction (5′–3′)	Sequence
circ_0004104	Forward	5′-AGACCTGTGACCTGGACAATG-3′
	Reverse	5′-GTGCACTTTGTGGCAAAGAA-3′
SPARC	Forward	5′-GGTATCTGTGGGAGCTAATC-3′
	Reverse	5′-CTGGTGGGGTCCTGGCACAC-3′
miR-328-3p	Forward	5′-CCTCTCTGCCCTTCCG-3′
	Reverse	5′-GAACATGTCTGCGTATCTC-3′
TRIM14	Forward	5′-GAGGTCGGAGCTTGTCGAG-3′
	Reverse	5′-TTCTTGGCTGAGTTTCTGCAC-3′
U6	Forward	5′-CTCGCTTCGGCAGCACA-3′
	Reverse	5′-AACGCTTCACGAATTTGCGT-3′
GAPDH	Forward	5′-AAGAAGGTGGTGAAGCAGGC-3′
	Reverse	5′-GTCAAAGGTGGAGGAGTGGG-3′

### Cyclization validation

RNA samples (2 μg) were incubated with 3 U/μg RNase R (Epicentre Technologies, Madison, WI, USA), and RNA levels were determined by RT-qPCR.

### Actinomycin D treatment

Transcription inhibitor Actinomycin D (2 mg/mL; Sigma, St. Louis, MO, USA) was added to the culture medium, and RNA levels were examined by RT-qPCR at specific time points.

### Oligonucleotides or plasmids transfection

Ectopic expression plasmid of circ_0004104 (circ_0004104), pLCDH-cir empty vector (vector), small interfering RNA against circ_0004104 (si-circ_0004104), negative control of siRNA (si-NC), TRIM14 overexpression plasmid (TRIM14) and empty vector (pcDNA) were purchased from Sangon Biotech, and mimics of miR-328-3p (miR-328-3p), miR-NC, inhibitor of miR-328-3p (anti-miR-328-3p) and anti-miR-NC were acquired from Genepharma (Shanghai, China). All oligonucleotides or plasmids were transfected into HUVECs with Lipofectamine 3000 reagent (Invitrogen).

### 3-(4,5-Dimethylthiazol-2-yl)-2,5-Diphenyltetrazolium Bromide (MTT) assay

At specific time points, HUVECs were incubated with MTT reagent (Sigma) for 4 h. Afterwards, a total of 200 μL dimethyl sulfoxide (DMSO; Sigma) was added to dissolve the formazan products after discarding cell supernatant. The absorbance (490 nm) was determined by the microplate reader (Bio-Rad, Hercules, CA, USA).

### Flow cytometry

HUVECs were simultaneously stained with Annexin V-fluorescein isothiocyanate (Annexin V-FITC) and propidium iodide (PI) of the Cell Apoptosis Detection Kit (Qiagen). The apoptotic percentage of HUVECs was evaluated by the flow cytometer (BD Biosciences, San Jose, CA, USA).

### Angiogenic capacity analysis via capillary-like network formation assay

HUVECs were plated onto Matrigel (BD Biosciences)-pre-coated 96-well cell culture plates (3 × 10^4^ cells/well). After culturing for 48 h, the average number of branches of each node was analyzed.

### Western blot assay

HUVECs were disrupted using whole cell lysis buffer (Beyotime, Shanghai, China). Protein samples (35 μg) were loaded onto sodium dodecyl sulfate–polyacrylamide gel electrophoresis (SDS-PAGE) and transferred onto polyvinylidene difluoride (PVDF) membrane (Millipore, Billerica, MA, USA). After sealing with 5% bovine serum albumin (BSA; Sangon Biotech), immunoblot assay was applied through incubating the membrane with the diluted primary antibodies and the horse radish peroxidase (HRP) conjugated secondary antibody (Abcam). Immuno-reactive signals were determined by the enhanced chemiluminescent (ECL) chromogenic substrate (Beyotime). The primary antibodies contained anti-Cleaved-caspase 3 (anti-Cleaved-casp3, ab32042, Abcam, Cambridge, MA, USA), anti-vascular endothelial growth factor A (anti-VEGFA, ab52917, Abcam), anti-TRIM14 (SAB1410027, Sigma) and anti-GAPDH (ab8245, Abcam).

### Enzyme-linked immunosorbent assay (ELISA)

The culture supernatant of HUVECs was collected to assess the release of tumor necrosis factor α (TNF-α) and interleukin 1β (IL-1β) using commercial Human TNF-α/IL-1β Quantikine ELISA Kit (R&D Systems, Minneapolis, MN, USA).

### Determination of cell oxidative stress

Cell oxidative stress was analyzed through measuring the production of superoxide dismutase (SOD) and malondialdehyde (MDA) using their corresponding commercial kits (Jiancheng Biotech, Nanjing, China).

### Bioinformatic analysis

StarBase database (http://starbase.sysu.edu.cn) was utilized to predict circ_0004104-miRNAs interactions and miR-328-3p-mRNAs interactions.

### Dual-luciferase reporter assay

The fragment of circ_0004104 or the 3′UTR fragment of TRIM14, including the miR-328-3p-binding sequence, was inserted into psiCHECK2 luciferase plasmid (Promega, Madison, WI, USA) to generate circ_0004104 wt and TRIM14 3′UTR wt. Meanwhile, mutated counterparts were constructed to generate circ_0004104 mut and TRIM14 3′UTR mut. HUVECs were seeded onto 12-well plates and co-transfected with luciferase plasmids and miR-NC or miR-328-3p. After 48-h transfection, the relative luciferase intensities were determined using the Dual-Luciferase Reporter Assay Kit (Promega).

### RNA immunoprecipitation (RIP) assay

RIP experiment was employed to confirm the binding relation between circ_0004104 and miR-328-3p with Magna RIP™ RNA-Binding Protein Immunoprecipitation Kit (Millipore). Cell extracts were prepared using RIP lysis buffer, and anti-Argonaute2 (anti-Ago2; Millipore) or anti-Immunoglobulin G (anti-IgG; Millipore)-pre-coated magnetic beads were incubated with cell lysates. The levels of enriched RNAs were measured by RT-qPCR.

### RNA-pull down assay

Cell lysates (2 μg) were incubated with 100 pmol Bio-miR-NC, Bio-miR-328-3p-mut or Bio-miR-328-3p-wt. The reaction mixture was then incubated with 100 μL agarose beads (Millipore) for 1 h. The retrieved RNAs were measured by RT-qPCR.

### Statistical analysis

All experiments were repeated for three times. Statistical analysis was carried out using GraphPad Prism 7.0 software (GraphPad, La Jolla, CA, USA). Data were represented as mean ± standard deviation (SD). The differences were analyzed by Student’s *t*-test (two groups) or one-way analysis of variance (ANOVA) (more than two groups). Differences were identified as statistically significant with the *P* value of less than 0.05.

## Results

### Characteristics of circ_0004104 in HUVECs

**Fig. 1:**
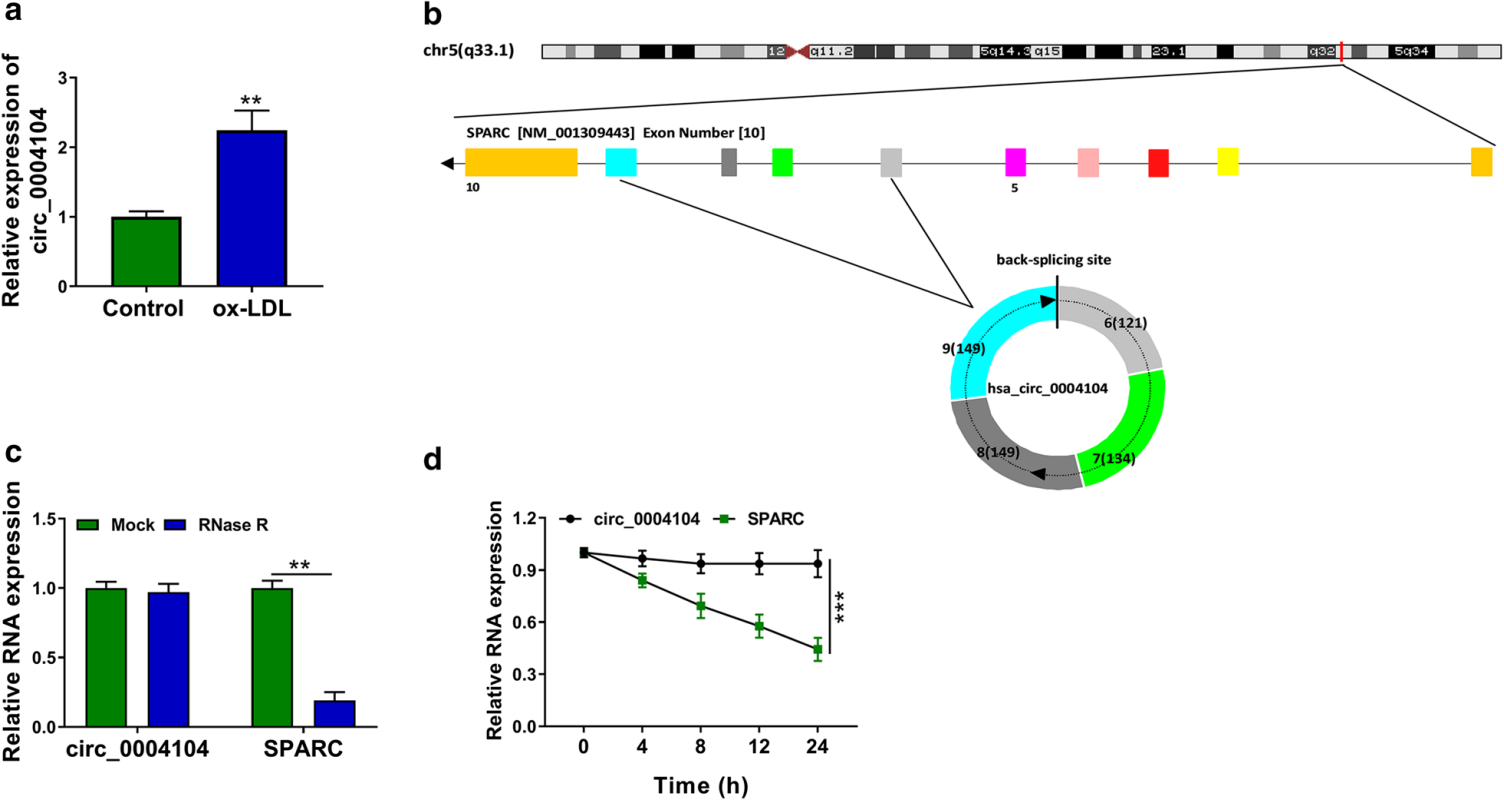
Characteristics of circ_0004104 in HUVECs. **a** RT-qPCR was applied to measure the level of circ_0004104 in HUVECs exposed to 100 μg/mL ox-LDL for 24 h or not. This experiment was performed for three times with three technical repetitions. **b** The chromosomal localization of circ_0004104 was shown. Circ_0004104 was derived from the back-splicing of the exon 6, 7, 8 and 9 in SPARC gene. **c** To test if circ_0004104 was a circular transcript, RNase R was utilized. RT-qPCR was performed to analyze the levels of circ_0004104 and its linear counterpart (SPARC) with or without RNase R digestion. This experiment was performed for three times. **d** The stability of circ_0004104 and SPARC was tested through adding transcription inhibitor Actinomycin D. RNA levels were examined by RT-qPCR. This experiment was performed for three times with three technical repetitions. **P < 0.01, ***P < 0.001. Student’s *t*-test was utilized to analyze the differences in all results of in this figure

Among several AS progression-associated circRNAs, including circ_0004104 [10], circ_0001879 [10], circ_0001445 [22], circ_0001599 [23], circ_0010283 [24] and circ_0007478 [25], we selected circ_0004104 for further analysis because it was the most significantly upregulated by ox-LDL (100 μg/mL, 24 h) in HUVECs (Additional file 1: Figure 1 and Fig. 1a). Circ_0004104 was derived from the back-splicing of exon 6–9 in SPARC gene (Fig. 1b). Circ_0004104 was resistant to RNase R relative to its linear counterpart SPARC (Fig. 1c), manifesting that circ_0004104 was indeed a circular transcript. With the treatment of transcriptional inhibitor Actinomycin D, the expression of circ_0004104 was almost unaffected (Fig. 1d), suggesting that circ_0004104 was more stable than its linear form SPARC in HUVECs.

### Circ_0004104 overexpression aggravates ox-LDL-induced dysfunction in HUVECs

**Fig. 2:**
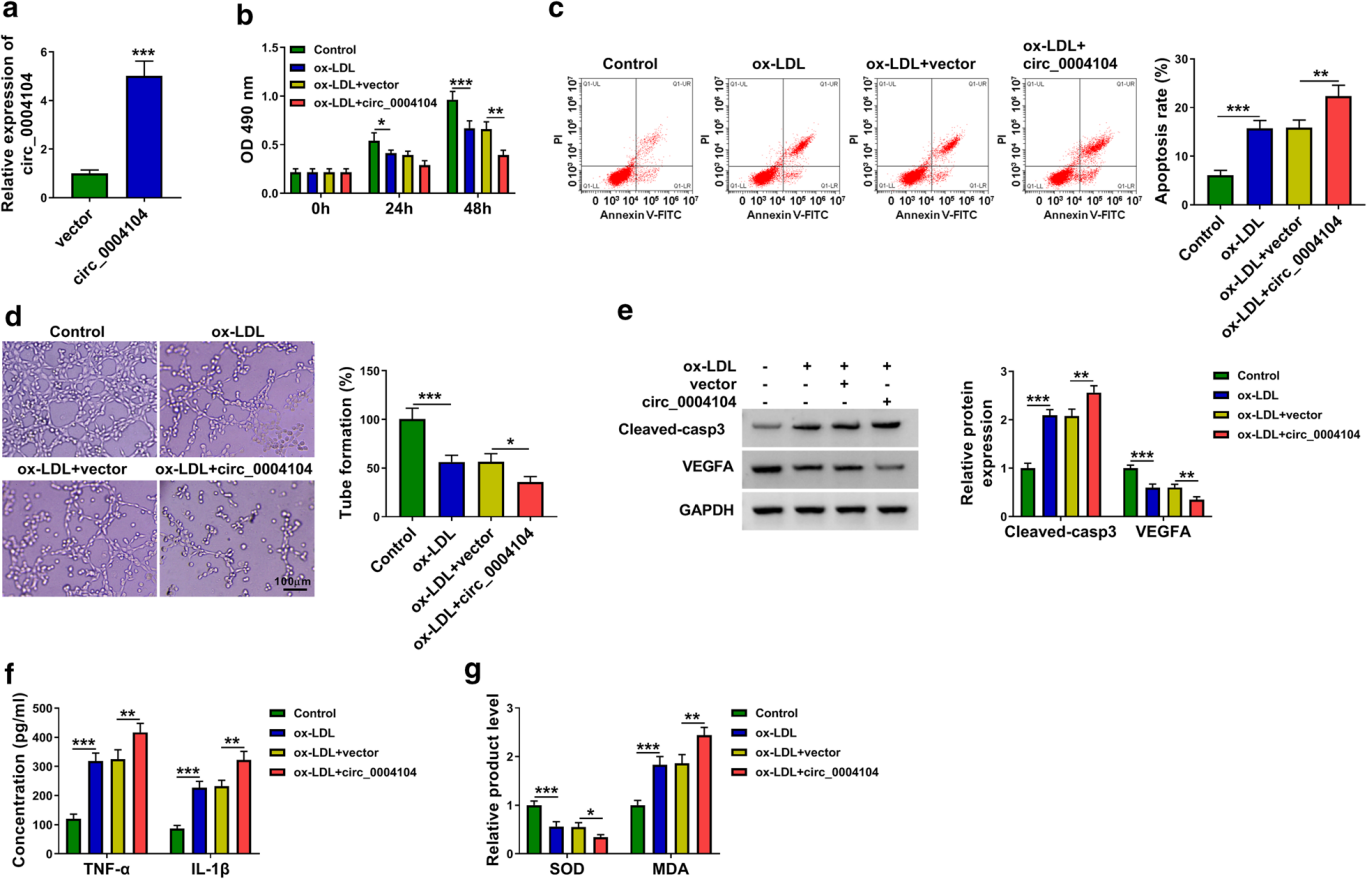
Circ_0004104 overexpression aggravates ox-LDL-induced dysfunction in HUVECs. **a** RT-qPCR was applied to assess the overexpression efficiency of circ_0004104 ectopic expression plasmid (circ_0004104) in HUVECs. **b**–**g** HUVECs were transfected with vector or circ_0004104 plasmid for 24 h prior to ox-LDL exposure (100 μg/mL; 24 h). This experiment was performed for three times with three technical repetitions. **b** MTT assay was conducted to analyze cell viability after 0 h, 24 h or 48 h. This experiment was performed for three times with six technical repetitions. **c** Cell apoptosis rate was assessed using flow cytometry. This experiment was performed for three times with three technical repetitions. **d** Capillary-like network formation assay was utilized to show capillary-like structure in four groups. This experiment was performed for three times with three technical repetitions. **e** Western blot assay was performed to measure the protein levels of apoptosis marker (Cleaved-casp3) and tube formation-associated marker (VEGFA) in HUVECs. This experiment was performed for three times. **f** ELISA was utilized to assess the levels of pro-inflammatory cytokines (TNF-α and IL-1β) to analyze cell inflammatory response. This experiment was performed for three times with three technical repetitions. **g** Cell oxidative stress was analyzed through measuring the levels of SOD and MDA using their matching kits. This experiment was performed for three times with three technical repetitions. *P < 0.05, **P < 0.01, ***P < 0.001. Student’s *t*-test was utilized to analyze the differences in (**a**), whereas one-way ANOVA was utilized to assess the differences in (**b**–**g**)

Transfection with circ_0004104 ectopic plasmid increased the level of circ_0004104 by five times in HUVECs (Fig. 2a). ox-LDL treatment suppressed cell viability and induced cell apoptosis, and these effects were further aggravated by the overexpression of circ_0004104 (Fig. 2b, c). Cell angiogenic ability was blocked by ox-LDL exposure, and circ_0004104 overexpression further inhibited the tube formation ability of HUVECs (Fig. 2d). Consistent with flow cytometry and capillary-like network formation assay, Western blot assay revealed that circ_0004104 overexpression further induced cell apoptosis and suppressed angiogenic ability in ox-LDL-treated HUVECs (Fig. 2e). ox-LDL exposure increased the release of TNF-α and IL-1β, and cell inflammatory response was further promoted with the accumulation of circ_0004104 (Fig. 2f). We also found that circ_0004104 overexpression aggravated ox-LDL-induced oxidative stress in HUVECs (Fig. 2g). Overall, circ_0004104 overexpression aggravated ox-LDL-induced dysfunction in HUVECs.

### ox-LDL-induced dysfunction is largely alleviated by the silencing of circ_0004104 in HUVECs

**Fig. 3:**
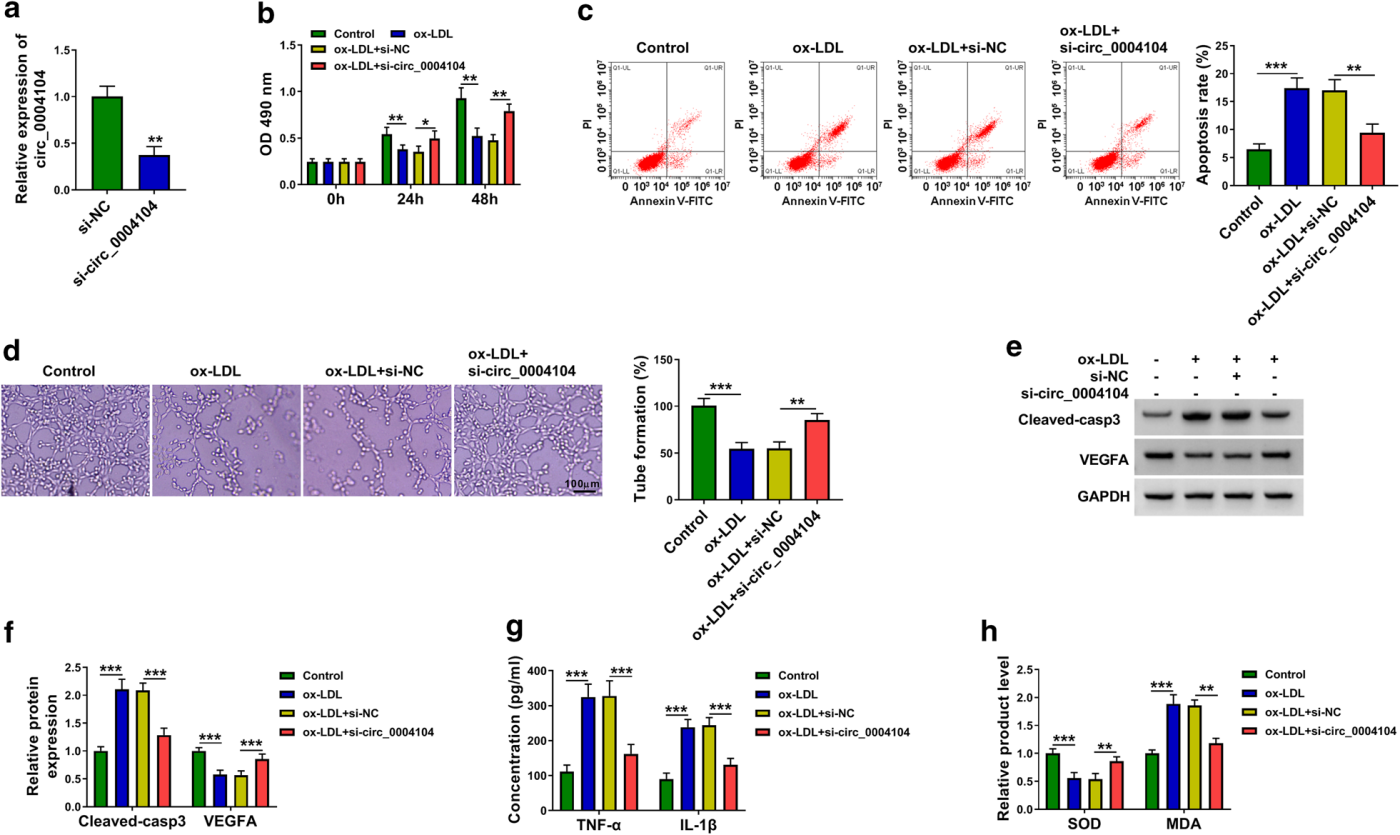
ox-LDL-induced dysfunction is largely alleviated by the silencing of circ_0004104 in HUVECs. **a** RT-qPCR was performed to evaluate the interference efficiency of si-circ_0004104 in HUVECs. This experiment was performed for three times with three technical repetitions. **b**–**h** HUVECs were divided into four groups: control, ox-LDL, ox-LDL + si-NC and ox-LDL + si-circ_0004101. **b** MTT assay was applied to analyze cell viability in different time points. This experiment was performed for three times with six technical repetitions. **c** The percentage of apoptotic HUVECs was assessed via flow cytometry. This experiment was performed for three times with three technical repetitions. **d** The ability of tube formation was analyzed through capillary-like network formation assay in vitro. This experiment was performed for three times with three technical repetitions. **e**, **f** The protein levels of Cleaved-casp3 and VEGFA were measured by Western blot assay. This experiment was performed for three times. **g** The concentrations of inflammation-associated cytokines (TNF-α and IL-1β) were detected by ELISA. This experiment was performed for three times with three technical repetitions. **h** The production of SOD and MDA was assessed using their matching kits. This experiment was performed for three times with three technical repetitions. *P < 0.05, **P < 0.01, ***P < 0.001. Student’s *t*-test was utilized to analyze the differences in **a**, whereas one-way ANOVA was utilized to assess the differences in (**b**–**h**)

RT-qPCR confirmed the high silencing efficiency of si-circ_0004104 in HUVECs (Fig. 3a). As shown in Fig. 3b, c, circ_0004104 knockdown recovered cell viability and suppressed cell apoptosis in HUVECs upon ox-LDL exposure. The silencing of circ_0004104 also largely rescued the ability of tube formation in ox-LDL-treated HUVECs (Fig. 3d). ox-LDL-induced up-regulation of Cleaved-casp3 and down-regulation of VEGFA were both attenuated by the addition of si-circ_0004104 in HUVECs (Fig. 3e, f). ox-LDL-induced inflammatory response and oxidative stress in HUVECs were largely alleviated by the silencing of circ_0004104 (Fig. 3g, h). Taken together, ox-LDL-induced dysfunction in HUVECs was partly based on the up-regulating circ_0004104.

### Circ_0004104 acts as miR-328-3p sponge in HUVECs

**Fig. 4:**
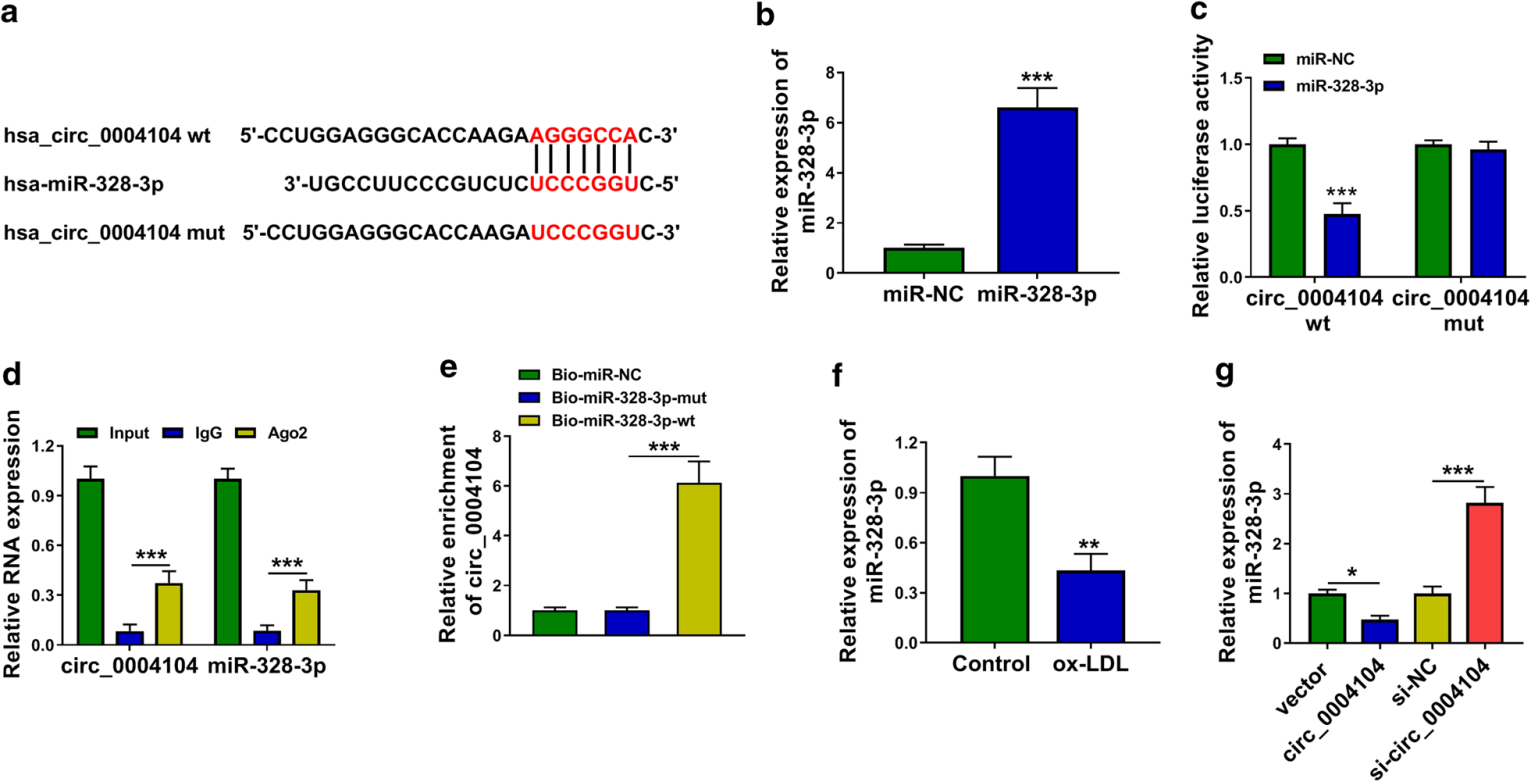
Circ_0004104 acts as miR-328-3p sponge in HUVECs. **a** The miR-328-3p-binding sites in circ_0004104 predicted by StarBase database were shown. **b** The overexpression efficiency of miR-328-3p was assessed via RT-qPCR. This experiment was performed for three times with three technical repetitions. **c** Dual-luciferase reporter assay was applied to verify the interaction between circ_0004104 and miR-328-3p as well as their binding sites. This experiment was performed for three times with three technical repetitions. **d** RIP assay was carried out to test if miR-328-3p was a target of circ_0004104 in HUVECs. This experiment was performed for three times with three technical repetitions. **e** The target relationship between circ_0004104 and miR-328-3p was tested by RNA-pull down assay. This experiment was performed for three times with three technical repetitions. **f** The expression of miR-328-3p in ox-LDL-treated HUVECs was measured by RT-qPCR. This experiment was performed for three times with three technical repetitions. **g** The regulatory relationship between circ_0004104 and miR-328-3p was tested. The expression of miR-328-3p was analyzed in HUVECs transfected with vector, circ_0004104, si-NC or si-circ_0004104 by RT-qPCR. This experiment was performed for three times with three technical repetitions. *P < 0.05, **P < 0.01, ***P < 0.001. Student’s *t*-test was utilized to analyze the differences in (**b**–**f**), whereas one-way ANOVA was utilized to assess the differences in (**g**)

The putative binding sites between miR-328-3p and circ_0004104 predicted by StarBase database were shown in Fig. 4a. Transfection with miR-328-3p mimics markedly up-regulated miR-328-3p expression in HUVECs (Fig. 4b). MiR-328-3p overexpression significantly reduced luciferase activity of wild-type circ_0004104 luciferase reporter plasmid (circ_0004104 wt) rather than mutant-type plasmid (circ_0004104 mut) (Fig. 4c), suggesting that circ_0004104 interacted with miR-328-3p via the predicted sites. The target relationship between miR-328-3p and circ_0004104 was also confirmed by RIP assay and RNA-pull down assay. ox-LDL exposure down-regulated the expression of miR-328-3p in HUVECs (Fig. 4f). A negative regulatory relationship between circ_0004104 and miR-328-3p was observed in HUVECs (Fig. 4g). These findings suggested that miR-328-3p was a target of circ_0004104.

### Circ_0004104 silencing attenuates ox-LDL-induced dysfunction in HUVECs partly through up-regulating miR-328-3p

**Fig. 5:**
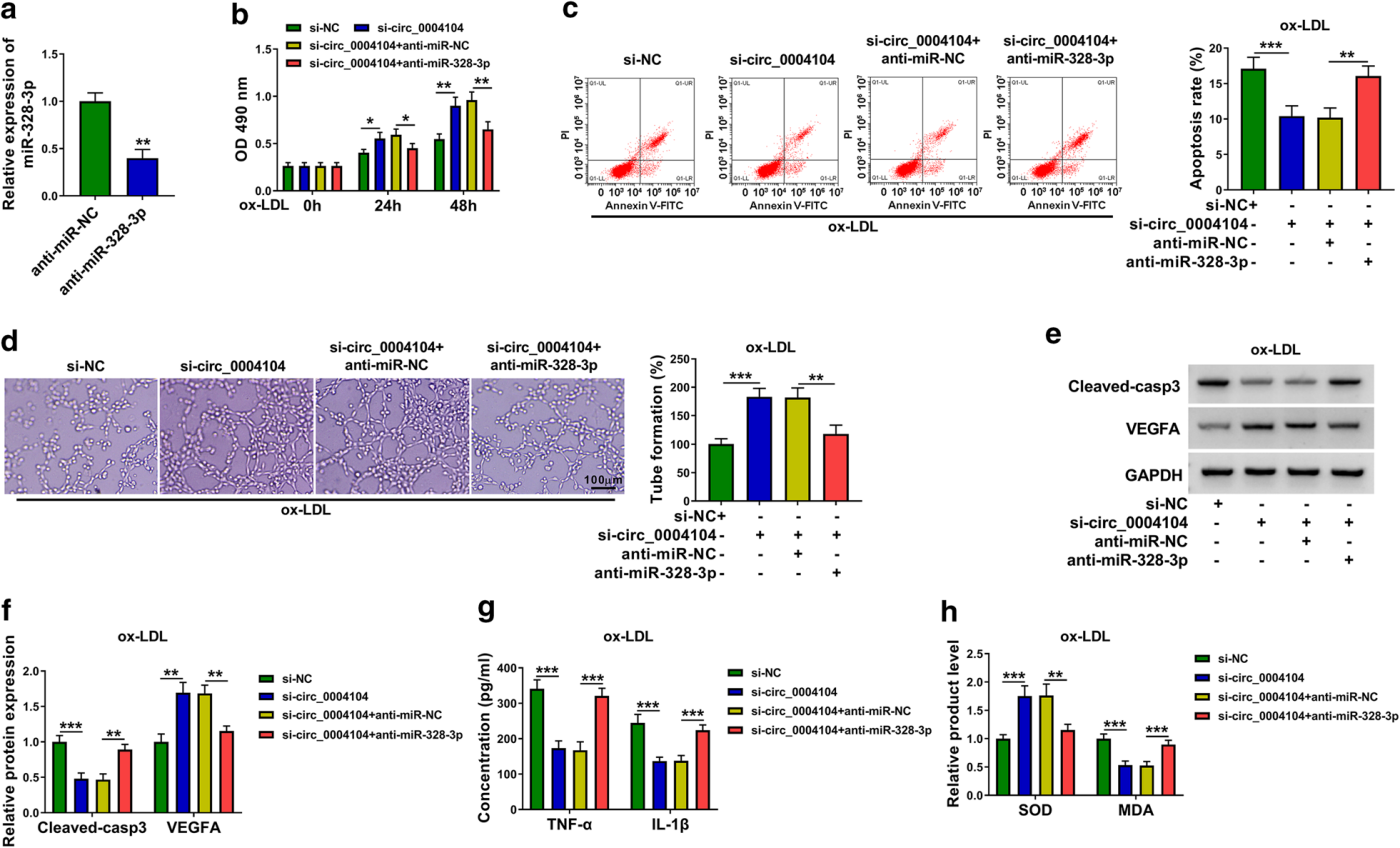
Circ_0004104 silencing attenuates ox-LDL-induced dysfunction in HUVECs partly through up-regulating miR-328-3p. **a** The interference efficiency of anti-miR-328-3p in HUVECs was tested by RT-qPCR. This experiment was performed for three times with three technical repetitions. **b** Cell viability was analyzed by MTT assay. This experiment was performed for three times with six technical repetitions. **c** Flow cytometry was carried out to analyze cell apoptosis rate in different groups. This experiment was performed for three times with three technical repetitions. **d** The angiogenic ability was analyzed using capillary-like network formation assay in vitro. This experiment was performed for three times with three technical repetitions. **e**, **f** Western blot assay was applied to analyze the levels of Cleaved-casp3 and VEGFA in transfected HUVECs. This experiment was performed for three times. **g** ELISA was utilized to assess the levels of TNF-α and IL-1β in the supernatant of HUVECs. This experiment was performed for three times with three technical repetitions. **h** The levels of SOD and MDA were detected using their matching commercial kits. This experiment was performed for three times with three technical repetitions. *P < 0.05, **P < 0.01, ***P < 0.001. Student’s *t*-test was utilized to analyze the differences in (**a**), whereas one-way ANOVA was utilized to assess the differences in (**b**–**h**)

Transfection with anti-miR-328-3p resulted in about 60% reduction in miR-328-3p level in HUVECs (Fig. 5a). MiR-328-3p silencing suppressed cell viability and angiogenic ability and induced cell apoptosis in circ_0004104-silenced HUVECs upon ox-LDL exposure (Fig. 5b–d). Consistently, Western blot assay revealed that miR-328-3p silencing overturned circ_0004104 knockdown-mediated effects in the expression of Cleaved-casp3 and VEGFA in ox-LDL-induced HUVECs (Fig. 5e, f). Circ_0004104 silencing protected HUVECs from ox-LDL-induced inflammation and oxidative, and these protective effects were overturned by the silencing of miR-328-3p (Fig. 5g, h). Overall, circ_0004104 knockdown protected HUVECs against ox-LDL-induced dysfunction partly through up-regulating miR-328-3p.

### TRIM14 is a downstream target of miR-328-3p in HUVECs

**Fig. 6:**
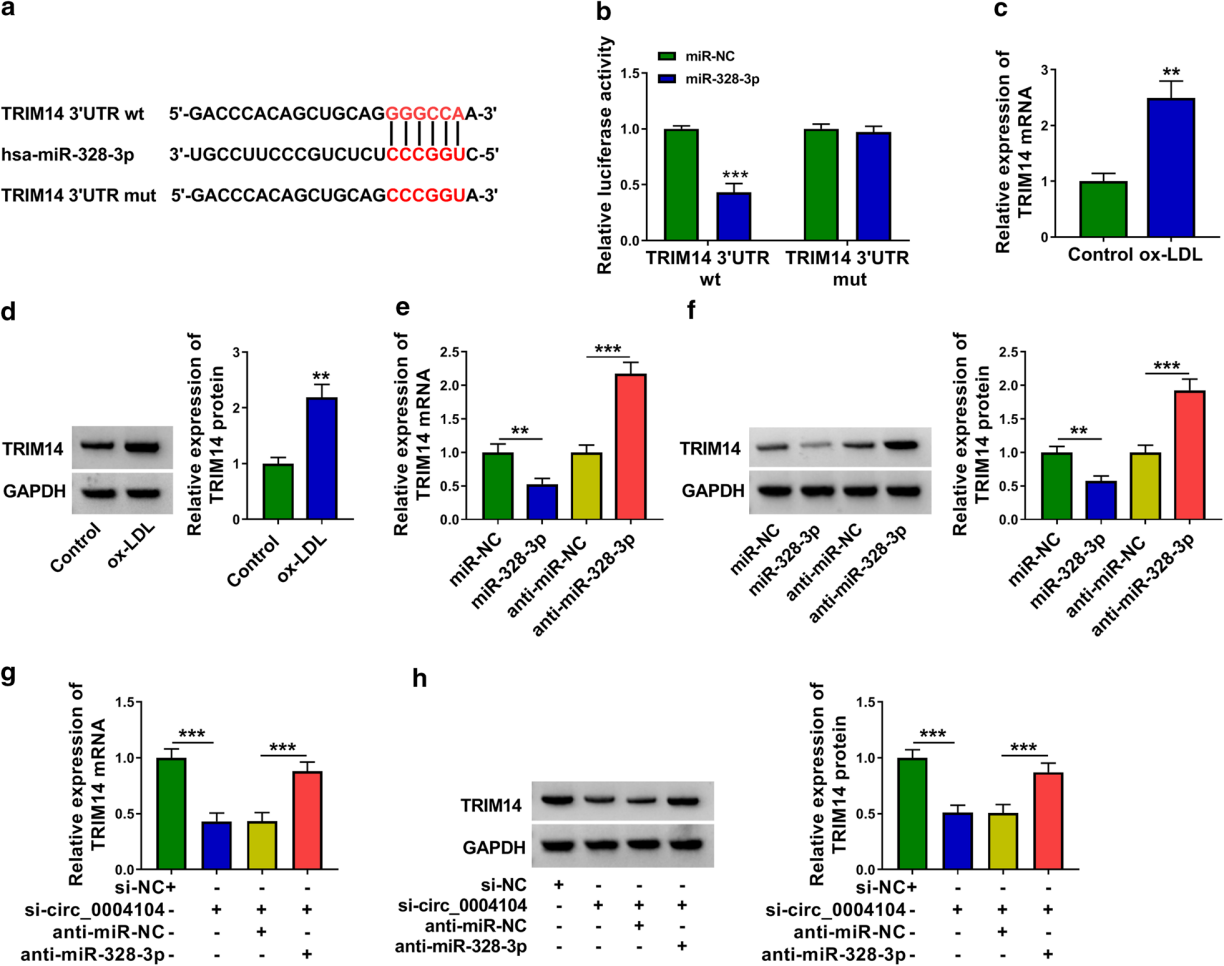
TRIM14 is a downstream target of miR-328-3p in HUVECs. **a** The interacted sites between miR-328-3p and TRIM14 3′UTR predicted by StarBase database were shown. **b** The binding relationship between miR-328-3p and TRIM14 was tested using dual-luciferase reporter assay. This experiment was performed for three times with three technical repetitions. **c**, **d** The expression of TRIM14 at mRNA and protein levels was measured in HUVECs exposed to ox-LDL or not by RT-qPCR and Western blot assay. RT-qPCR was applied for three times with three technical repetitions, and Western blot assay was conducted for three times. **e**, **f** The mRNA and protein expression of TRIM14 was analyzed in HUVECs with the overexpression or knockdown of miR-328-3p by RT-qPCR and Western blot assay. RT-qPCR was applied for three times with three technical repetitions, and Western blot assay was conducted for three times. **g**, **h** HUVECs were transfected with si-circ_0004104 alone or together with anti-miR-328-3p. The mRNA and protein levels of TRIM14 were detected by RT-qPCR and Western blot assay. RT-qPCR was applied for three times with three technical repetitions, and Western blot assay was conducted for three times. **P < 0.01, ***P < 0.001. Student’s *t*-test was utilized to analyze the differences in **b**–**d**, whereas one-way ANOVA was utilized to assess the differences in **e**–**h**

The potential binding sequence between miR-328-3p and TRIM14 predicted by StarBase was shown in Fig. 6a. Luciferase activity of wild-type luciferase reporter plasmid (TRIM14 3′UTR wt) was notably reduced with the overexpression of miR-328-3p (Fig. 6b), suggesting that TRIM14 was a target of miR-328-3p in HUVECs. ox-LDL exposure up-regulated the expression of TRIM14 at both mRNA and protein levels (Fig. 6c, d). The negative regulatory relationship between miR-328-3p and TRIM14 was observed in HUVECs (Fig. 6e, f). Subsequently, we analyzed the regulation among circ_0004104, miR-328-3p and TRIM14 in HUVECs. Circ_0004104 interference reduced the mRNA and protein expression of TRIM14 partly by up-regulating miR-328-3p in HUVECs (Fig. 6g, h). Overall, circ_0004104 positively regulated TRIM14 expression by sponging miR-328-3p in HUVECs.

### MiR-328-3p overexpression alleviates ox-LDL-induced dysfunction in HUVECs partly through reducing TRIM14 expression

**Fig. 7:**
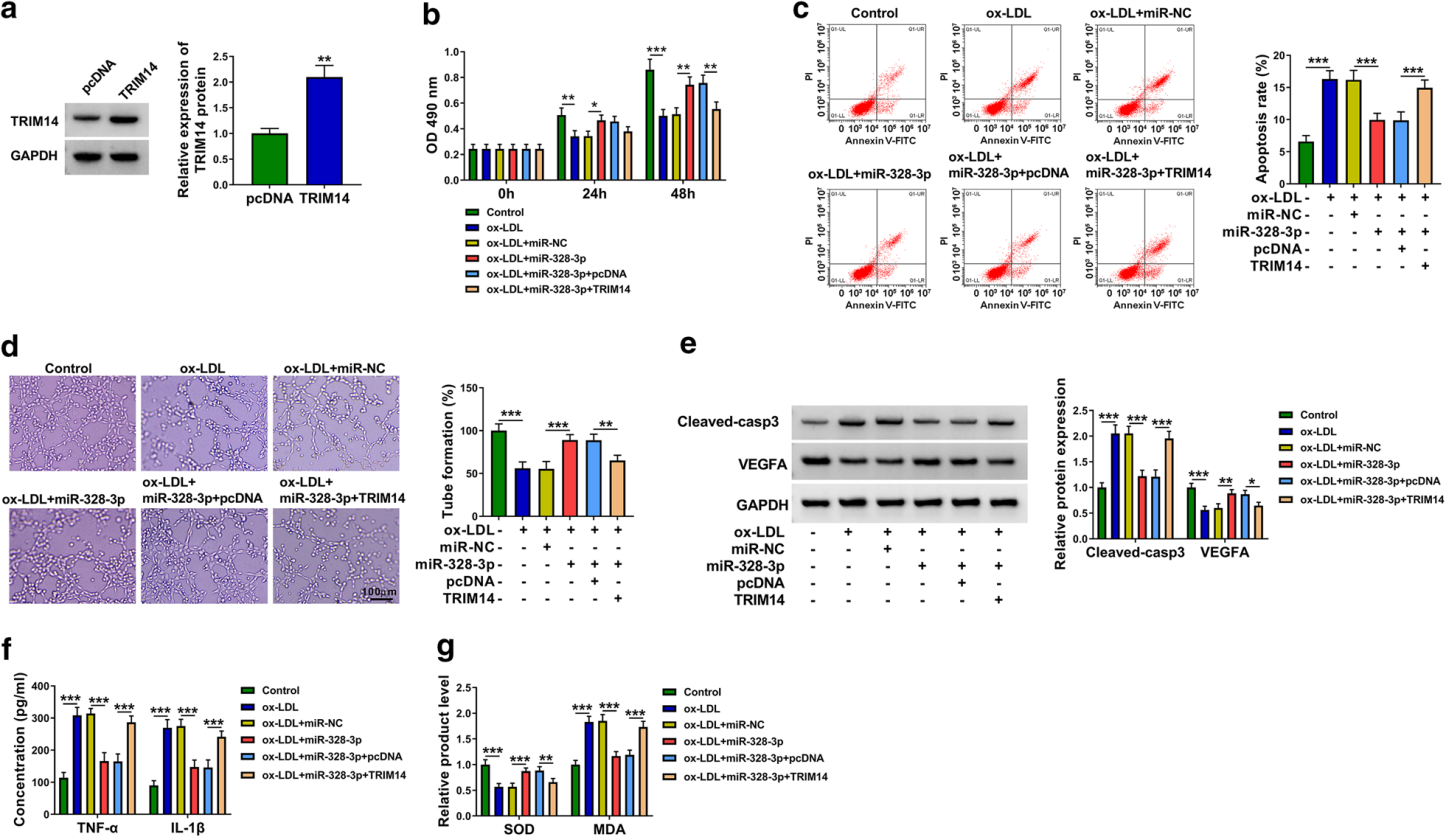
MiR-328-3p overexpression alleviates ox-LDL-induced dysfunction in HUVECs partly through reducing TRIM14 expression. **a** The overexpression efficiency of TRIM14 plasmid was tested by Western blot assay. This experiment was performed for three times. **b**–**g** HUVECs were divided into six groups: control, ox-LDL, ox-LDL + miR-NC, ox-LDL + miR-328-3p, ox-LDL + miR-328-3p + pcDNA and ox-LDL + miR-328-3p + TRIM14. **b** Cell viability in indicated time points was measured by MTT assay. This experiment was performed for three times with six technical repetitions. **c** Flow cytometry was carried out to analyze cell apoptosis rate in transfected HUVECs. This experiment was performed for three times with three technical repetitions. **d** Capillary-like network formation assay was performed to show capillary-like structure in six groups. This experiment was performed for three times with three technical repetitions. **e** The protein levels of Cleaved-casp3 and VEGFA were detected via Western blot assay. This experiment was performed for three times. **f** The levels of pro-inflammatory cytokines, including TNF-α and IL-1β, were analyzed by ELISA. This experiment was performed for three times with three technical repetitions. **g** The production of SOD and MDA was evaluated using their corresponding kits. This experiment was performed for three times with three technical repetitions. *P < 0.05, **P < 0.01, ***P < 0.001. Student’s *t*-test was utilized to analyze the differences in (**a**), whereas one-way ANOVA was utilized to assess the differences in (**b**–**g**)

Western blot assay showed that the transfection efficiency of TRIM14 plasmid was high in HUVECs (Fig. 7a). MiR-328-3p overexpression protected HUVECs from ox-LDL-induced dysfunction of HUVECs (Fig. 7b–g). The addition of TRIM14 plasmid suppressed cell viability and angiogenesis whereas induced the apoptosis in miR-328-3p-overexpressed HUVECs upon ox-LDL exposure (Fig. 7b–d). TRIM14 overexpression also up-regulated Cleaved-casp3 expression and reduced VEGFA expression (Fig. 7e), which further demonstrated that TRIM14 overexpression reversed miR-328-3p overexpression-mediated effects in the apoptosis and angiogenesis of HUVECs upon ox-LDL exposure. The accumulation of TRIM14 promoted the inflammatory response and oxidative stress again in miR-328-3p-overexpresed HUVECs upon ox-LDL (Fig. 7f, g). Overall, miR-328-3p overexpression protected HUVECs against ox-LDL-induced dysfunction partly through reducing TRIM14 expression.

## Discussion

AS results in severe cardio/cerebral-vascular disorders, containing coronary heart disease and stroke [27]. The phenotypic transformation of endothelial cells is an important induction factor in AS initiation and progression [28]. Hence, it is essential to uncover the crucial molecules involved in the phenotypic transformation of endothelial cells. Dysregulated circRNAs have been associated with AS progression by previous studies [21, 29]. For instance, circ_0044073 was highly expressed in AS, and it promoted the proliferation and invasion of vascular smooth muscle cells and vascular endothelial cells through sponging miR-107 in AS [30]. Zhang et al. demonstrated that circ-PTPRA contributed to AS development through up-regulating SP1 via sponging miR-636 [31]. As for circ_0004104, Wang et al. found that circ_0004104 was highly expressed in coronary artery disease patients compared with controls, and circ_0004104 was identified as a novel bio-marker for the diagnosis of coronary artery disease [10]. However, the expression pattern and role of circ_0004104 in the pathogenesis of AS remain to be disclosed. Here, we established AS cell model through exposing HUVECs to ox-LDL. Circ_0004104 abundance was markedly up-regulated in ox-LDL-exposed HUVECs relative to un-treated HUVECs. Through using exonuclease RNase R and transcriptional inhibitor Actinomycin D, we found that circ_0004104 was a stable circular transcript. ox-LDL exposure restrained cell viability and tube formation ability and induced the apoptosis, inflammation and oxidative stress status of HUVECs. To analyze the role of circ_0004104 in AS, we overexpressed or silenced circ_0004104 in AS cell model. Circ_0004104 overexpression aggravated ox-LDL-induced injury in HUVECs, whereas ox-LDL-induced damage in HUVECs was largely alleviated by the silencing of circ_0004104, which demonstrated that ox-LDL induced the injury of HUVECs partly through up-regulating circ_0004104.

“MiRNA sponge” mechanism is an important way by which circRNAs function in human diseases [26, 32]. For example, circ_0010283 accelerated cell viability and motility of ox-LDL-treated vascular smooth muscle cells by sponging miR-370-3p and up-regulating HMGB1 [24]. Circ-PRMT5 contributed to gastric cancer development through up-regulating MYC via sponging miR-145 and miR-1304 [33]. To investigate the molecular mechanism by which circ_0004104 functioned in ox-LDL-induced HUVECs, we analyzed circ_0004104-miRNA interactions using bioinformatic database StarBase. MiR-328-3p was predicted as a possible target of circ_0004104 via StarBase database, and their target relation was then confirmed by dual-luciferase reporter assay, RIP assay and RNA-pull down assay. MiR-328-3p was identified as a tumor suppressor in several malignancies through modulating its downstream genes and signal pathways [34–36]. Furthermore, Xing et al. demonstrated that miR-328-3p restrained the proliferation and cell cycle progression of pulmonary artery smooth muscle cells [37]. Qin et al. demonstrated that miR-328-3p alleviated ox-LDL-induced injury in vascular endothelial cells through reducing FOXO4 abundance in AS [14]. We found that ox-LDL exposure reduced the level of miR-328-3p in HUVECs. In addition, we found that miR-328-3p was negatively regulated by circ_0004104 in HUVECs. To explore if circ_0004104 functioned through sponging miR-328-3p, we transfected HUVECs with si-circ_0004104 alone or together with anti-miR-328-3p prior to ox-LDL exposure to perform rescue experiments. The results uncovered that circ_0004104 silencing attenuated ox-LDL-mediated injury in HUVECs partly through up-regulating miR-328-3p.

MiRNAs could regulate gene expression through repressing translation or degrading target mRNAs [12]. StarBase database was utilized to explore the downstream molecules of miR-328-3p. TRIM14 was predicted to be a candidate target of miR-328-3p, and their intermolecular interaction was subsequently verified by dual-luciferase reporter assay. TRIM14 was identified as an oncogene in a variety of cancers. For instance, TRIM14 accelerated the migration and invasion abilities of colorectal cancer cells through regulating SPHK1/STAT3 signaling [38]. TRIM14 elevated the drug resistance of glioma cells through regulating Wnt/β-catenin signaling [39]. Huang et al. found that TRIM14 accelerated the activation of endothelium via activating NF-κB pathway [21]. We found that ox-LDL exposure up-regulated the expression of TRIM14 at mRNA and protein levels. We also found that TRIM14 was negatively regulated by miR-328-3p in HUVECs. We found that circ_0004104 positively regulated TRIM14 expression through acting as miR-328-3p sponge. To explore if miR-328-3p regulated the biological phenotypes of HUVECs via targeting TRIM14, we performed compensation experiments. The results revealed that miR-328-3p protected HUVECs against ox-LDL-mediated damage in HUVECs partly through down-regulating TRIM14.

## Conclusions

In conclusion, our study demonstrated that circ_0004104 contributed to ox-LDL-induced injury of HUVECs partly through targeting miR-328-3p/TRIM14 axis. Blockage of circ_0004104 might be a potential strategy to attenuate the abnormal phenotypes of vascular endothelial cells in AS.

## Supplementary Information

**Additional file 1: Figure 1**. The expression of AS progression-associated circRNAs in HUVECs upon ox-LDL exposure. RT-qPCR was applied to analyze the levels of circ_0004104, circ_0001879, circ_0001445, circ_0001599, circ_0010283 and circ_0007478 in HUVECs induced by ox-LDL. This experiment was performed for three times with three technical repetitions. *P < 0.05, **P < 0.01. Student’s t-test was utilized to analyze the differences.

## Data Availability

The datasets used and/or analysed during the current study are available from the corresponding author on reasonable request.
