# Supplementary figures and images for: Circ_0004104 knockdown alleviates oxidized low-density lipoprotein-induced dysfunction in vascular endothelial cells through targeting miR-328-3p/TRIM14 axis in atherosclerosis
Source: BMC Cardiovasc Disord. 2021 Apr 23;21:207. doi: 10.1186/s12872-021-02012-7 (PMC8066471; doi:10.1186/s12872-021-02012-7)

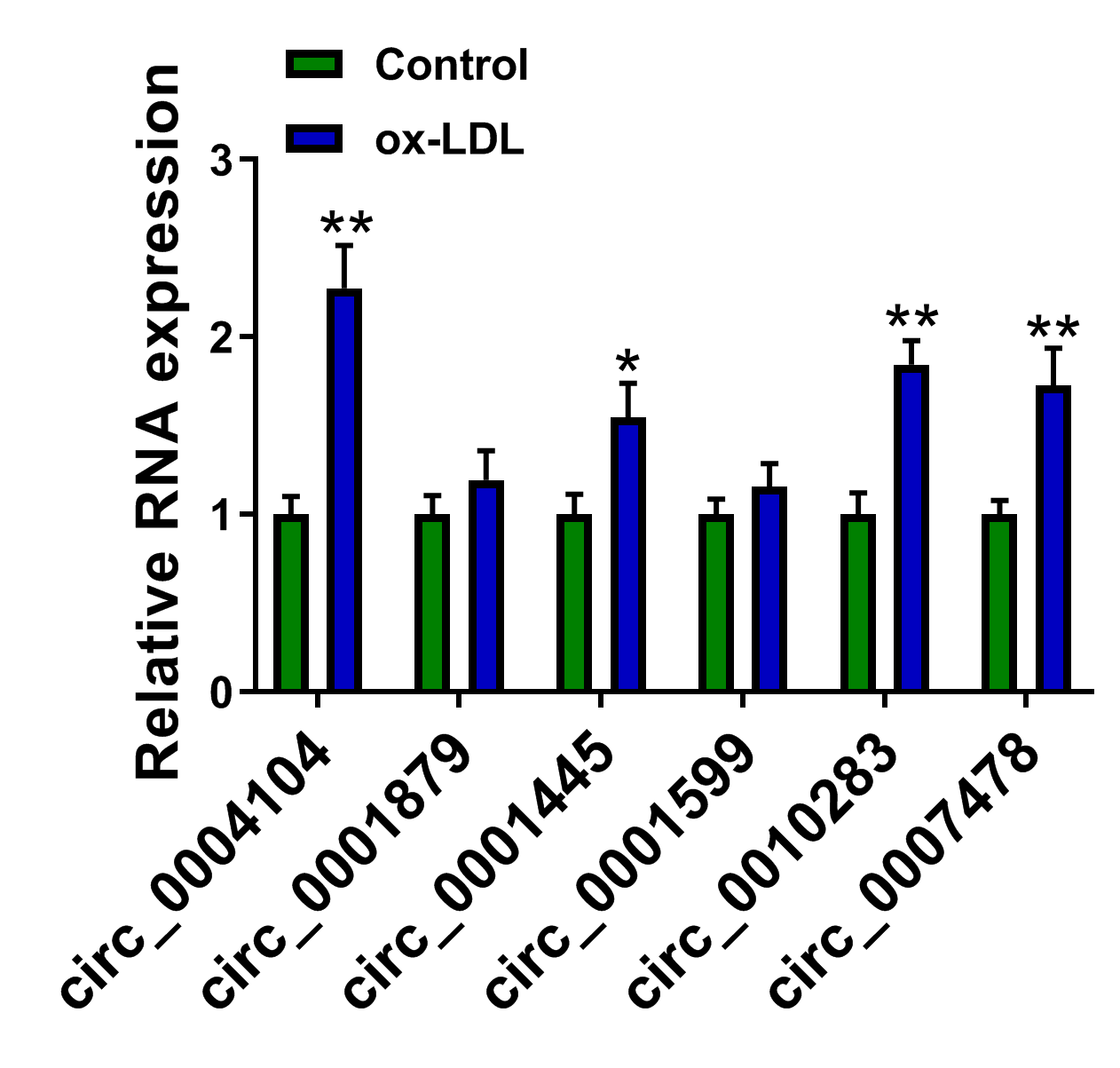

Supplement: Supplementary file 1 — Additional file 1: Figure 1. The expression of AS progression-associated circRNAs in HUVECs upon ox-LDL exposure. RT-qPCR was applied to analyze the levels of circ_0004104, circ_0001879, circ_0001445, circ_0001599, circ_0010283 and circ_0007478 in HUVECs induced by ox-LDL. This experiment was performed for three times with three technical repetitions. *P < 0.05, **P < 0.01. Student’s t-test was utilized to analyze the differences. [file 12872_2021_2012_MOESM1_ESM.tif]
